# The Effectiveness and Efficiency of Disease Management Programs for Patients with Chronic Diseases

**DOI:** 10.5539/gjhs.v5n2p27

**Published:** 2012-11-27

**Authors:** Akinori Hisashige

**Affiliations:** 1 Institute of Healthcare Technology Assessment, Tokushima, Japan

**Keywords:** disease management, effectiveness, efficiency, process, health services

## Abstract

**Objective:**

Disease management (DM) approach is increasingly advocated as a means of improving effectiveness and efficiency of healthcare for chronic diseases. To evaluate the evidence on effectiveness and efficiency of DM, evidence synthesis was carried out.

**Methods:**

To locate eligible meta-analyses and systematic reviews, we searched Medline, EMBASE, the Cochrane Library, SCI-EXPANDED, SSCI, A&HCI, DARE, HTA and NHS EED from 1995 to 2010. Two reviewers independently extracted data and assessed a study quality.

**Results:**

Twenty-eight meta-analyses and systematic reviews were included for synthesizing evidence. The proportion of articles which observed improvement with a reasonable amount of evidence was the highest at process (69%), followed by health services (63%), QOL (57%), health outcomes (51%), satisfaction (50%), costs (38%) and so on. As to mortality, statistically significant results were observed only in coronary heart disease. Important components in DM, such as a multidisciplinary approach, were identified.

**Conclusion:**

The evidence synthesized shows considerable evidence in the effectiveness and efficiency of DM programs in process, health services, QOL and so on. The question is no longer whether DM programs work, but rather which type or component of DM programs works best and efficiently in the context of each healthcare system or country.

## 1. Introduction

The growing burden of chronic diseases, such as coronary heart disease, diabetes mellitus, depression, asthma, cancer, and so on, has contributed to increasing healthcare costs in the past decades all over the world (WHO, 2005; Adeyl et al., 2007). The management of healthcare for persons with chronic diseases has advanced substantially in recent decades, yet these issues remain deficient. To improve systematically the quality and efficiency of healthcare for chronic diseases are critical problems for healthcare decision-making (Wagner et al., 2002).

The disease management (DM) approach is increasingly advocated as a means of improving effectiveness and efficiency of healthcare for chronic diseases (Hunter et al., 1997). DM generally refers to a systematic population-based approach emphasizing coordinated and comprehensive care along the continuum of disease and across the health care delivery system (Epstein et al., 1997; Ellrodt et al., 1997). DM programs are complex and have many components, which are a combination of patient education, provider use of practice guidelines, appropriate education, and supplies of drugs and ancillary services (Hunter et al., 1997).

DM appealed to healthcare decision maker keen to contain costs and improve health outcome. Initially, DM programs were mainly extension services offered by the US pharmaceutical companies, but evolved into disease-specific programs and more recently into comprehensive condition management programs (Walker et al., 2002; Shelton, 2002). Moreover, DM programs have evolved and been disseminated into other countries and organizations, such as the EU (Department of Health, 2002; Busse, 2004; Singh, 2008), Canada (Wong et al., 2004) and WHO (2002).

Numerous studies have been carried out to evaluate the impact of DM programs. However, the quality of these studies has varied very widely and the effectiveness of DM programs has remained undetermined (Walker et al., 2002; Linden et al., 2005). Moreover, recent large randomized trials of Medicare-coordinated care demonstration for DM under a fee-for-service context in the US failed to demonstrate effectiveness or cost reduction (Peikes et al., 2009). Those results have evoked a lot of controversies on the value of DM programs in the US (Ayanian, 2009) and have led to a focus on alternative approaches such as medical home (Rosental, 2008), care coordination (Boult et al., 2010), and transitional care (Naylor et al., 2004). However, while firm evidence on effectiveness and /or efficiency for these approaches is still lacking, recent analyses of the method of Medicare demonstration projects suggested that well-targeted DM efforts can be cost-effective (Brown, 2009).

Therefore, to examine and summarize the existing evidence on effectiveness and efficiency of diverse DM programs, as well as DM's applicability to a healthcare system is extremely valuable to the formulation of future healthcare decision making and the exploration of possibilities for new approaches. The author has examined these issues before (Velasoc-Garrido et al., 2003), and indicated that most of the DM programs evaluated have been shown to improve the management of diseases, although there is insufficient evidence on mortality reduction, as the final outcome, and cost-effectiveness. Since then, numerous meta-analyses and systematic reviews of DM in several disease areas have been expanded and accumulated rapidly. This article critically evaluates and synthesizes the evidence on effectiveness and efficiency of DM programs from meta-analyses and/or systematic reviews available.

## 2. Methods

Since there is no consensus about the definition of disease management, we have done a systematic literature search for evidence from meta-analyses and/or systematic reviews relative to the effectiveness and efficiency of DM programs for chronic diseases, by using a broad definition of disease management with several key components (Hunter et al., 1997; Epstein et al., 1997; Ellrodt et al., 1997), as is mentioned before. The inclusion criteria for relevant articles were as follows: a reference to the definition of disease management, inclusion of more than one component of disease management programs, satisfaction for minimum requirement for meta-analyses or systematic reviews (e.g., explicit question and search strategy), and outcomes measures (e.g., patient outcomes or costs). As an exception, articles relative to the Chronic Care Model (Bodenheimer et al., 2002) were included, even if they did not refer to disease management. Articles with only specific and single intervention (e.g., self-management, case management, etc), or no outcome measure, and narrative reviews were excluded.

To identify articles as meta-analyses and/or systematic reviews of DM programs, we conducted systematic literature searches in several databases between January 1995 and May 2010. The former report confirmed that there was no relevant article before 1995. Firstly, as a preliminary search, we conducted a search in Medline with the following key words: ‘disease management’ and, ‘meta-analysis’ or ‘systematic review’. Ninety-three articles were identified as meta-analyses or systematic reviews. Their contents were examined to construct a further literature search strategy and 8 key outcome items for data extraction: mortality, health outcomes, process, quality of life (QOL), satisfaction, knowledge or life-style change, health services, and costs.

Based on this result, the following databases were searched: Medline, EMBASE, SCI-EXPANDED, SSCI, A&HCI, Cochrane Library (Cochran Reviews, DARE, HTA, NHS EED). We constructed a search strategy by using combinations of the following keywords: “disease management or disease management programs”, “comprehensive multidisciplinary program”, “case management or case management program”, “care management or care management program”, “chronic disease”, “meta-analysis”, and “systematic review”.

Titles and abstracts of articles were reviewed for relevance according to the inclusion criteria by two reviewers, who are experts in health technology assessment with experience over 20 years, and, if potentially relevant, we retrieved the full-text article. The items of information were extracted and synthesized. The initial search strategy identified 2304 references. Independently, two researchers reviewed both all titles and abstracts simultaneously. After removing articles, which dealt with irrelevant topics, acute diseases, and primary studies, we accepted 102 articles for further screening as potentially eligible ones and reviewed their full texts. Twenty-eight articles met our inclusion criteria: 20 were meta-analyses (Gohler et al., 2006; Roccaforte et al., 2005; Wellan et al., 2005; Phillips et al., 2005; Gonseth et al., 2004; McAlister et al., 2001a, 2001b; Lemmens et al., 2009; Peytremann-Bridevaux et al., 2008; Adams et al., 2007; Taylor et al., 2005; Sin et al., 2003; Knight et al., 2005; Noris et al., 2002; Neumeyer-Gromen et al., 2004; Badamgarav et al., 2003a, 2003b; Tsai et al., 2005; Krause, 2005; Weingarten et al., 2002) and the remaining 8 were qualitative systematic reviews (Yu et al., 2006; Jerant et al., 2005; Ara, 2004; Maciejewski et al., 2009; Steuten et al., 2009; Niesink et al., 2007; Steuten et al., 2007; Ofman et al., 2004). Articles excluded had the following characteristics: narrow focus on self-management, case management, care management, or health promotion, insufficient search strategy, recommendation oriented, annotated bibliography and narrative reviews.

A preliminary review was done to decide key outcome items, which were relevant for evaluating disease management programs. The following 8 items were identified: mortality, health outcomes (e.g., morbidity, disability and function), process (e.g., compliance or adherence to guidelines), quality of life, satisfaction, knowledge or life-style, health services (e.g., hospitalization or admission), and costs. Data on 8 key outcome items were extracted by two researchers, independently. Discrepancies were resolved by consensus development between them.

The quality of meta-analyses and systematic reviews were explicitly assessed according to the guide developed by the Evidence-Based Medicine Working Group (Oxman et al., 2002). Two items (i.e., validity and contents of results) and their 7 sub-items among three items in this guide were used to assess the quality of these analyses and reviews. The item related to applicability was excluded, since this item mainly depended on the context of each user. The proportion of sub-items satisfied by each paper was more than 70%, although the sub-item for precision of results was not applicable to qualitative reviews.

The data obtained were analyzed and presented according to the following items: the definition and components of DM, the effectiveness and efficiency of DM programs, the features of interventions in DM programs and economics of DM programs. This study was carried out between June 2010 and April 2011.

## 3. Results

### 3.1 Definition and Components of Disease Management

Table 1 shows the definitions of DM programs cited among all 28 articles evaluated. Although there was something similar among them, there existed no common definition of DM, and a considerable variation was observed, strictly speaking. While seven articles had their own definitions (Gohler et al., 2006; Whellan et al., 2005; Phillips et al., 2005; Maciejewski et al., 2009; Nori et al., 2002, Neumeyer-Gromen et al., 2004; Badamgarav et al., 2003a), four articles (Gonseth et al., 2004; Niesink et al., 2007; Weingarten et al., 2002; Ofman et al., 2004) adopted the definition of Weingarten (2002) and four articles (Lemmens et al., 2009; Steuten et al., 2009; 2007; Krause, 2005) adopted that of the Disease Management Association America (2003). Three articles (Jerant et al., 2005; Ara, 2004; Knight et al., 2005) referred to the same definition of Epstein (Epstein et al., 1996). Also, three articles (Yu et al., 2006; Peytremann-Bridevaux et al., 2008; Badamgarav et al., 2003b) adopted that of Ellrodt (1997) and two articles (McAlister et al., 2001b; Sin et al., 2003) that of Hunter (1997). One specific definition of the chronic care model was mentioned in three articles (Adams et al., 2007; Steuten et al., 2009; Tsai et al., 2005). This definition did not use the expression of DM, but what it specified could be classified as DM in a broad sense. On the other hand, there was no explicit statement about the definition in three articles (Roccaforte et al., 2005; McAlister et al., 2001a; Taylor et al., 2005).

**Table 1:** Working definition of disease management (DM) and literature search

Article	Working definition	Databases and search strategy	Selected research and criteria
**CHD**			
***Meta-analysis***			
Göhler 2006	DM programs focus on disease education for the patient and continuing support after hospital discharge	Databases: Medline (1966~2005) Key words: congestive heart failure, disease management program, case management, early intervention, clinical protocol, patient care planning, nurse led clinics, home care services, etc	36 studies included (36 RCTs) Inclusion criteria: RCT, endpoints (all-cause mortality and/or all-cause hospitalization), comparing DM with standard care, at least 3 months of follow-up
Roccaforte 2005	No explicit statement (only the words: comprehensive DM program)	Databases: Medline, EMBASE, CINAHL, Cochrane (1980~2004) Key words: disease management, case management, comprehensive health care, health service research, health service costs, etc	33 studies included (RCTs) Inclusion criteria: out patient setting, comprehensive DM program, comparison with usual care, hospitalization rate, mortality
Whellan 2005	DM is viewed as a means to increase the use of evidence-based therapies, improve patient education, and decrease resource usage.	Databases: Medline (1966~2003) Key words: case management, comprehensive health care, health service research, home care services, clinical protocol, etc	19 studies included (RCTs) Inclusion criteria: randomized controlled trial
Phillips 2005	DM protocols employed a variety of interventions, with or without components for hospital discharge planning and widely differing strategies for post-discharge care.	Databases: Medline, EMBASE, Cochrane (1966~2004) Key words: multidisciplinary care, disease management, patient education, social work, case management, comprehensive discharge planning, etc	6 studies included (RCTs) Inclusion criteria: randomized allocation of at least 100 patients, clearly defined protocol, the addition of specialist heart failure nurses, heart failure clinics
Gonseth 2004	DM is an intervention designed to manage heart failure (HF) and reduce hospital re-admissions using a systematic approach to care and potentially employing multiple treatment modalities.	Databases: Medline, EMBASE, Cochrane (1966~2003) Key words: guidelines, clinical pathways, protocols, algorism, care plans, quality improvement activities, patient support and education	54 studies included (27 RCTs, 27 CTs) Inclusion criteria: controlled studies assessing DM programs targeted, among others, at patients aged equal or more than 65 years with principal or secondary diagnosis (with specific exclusion criteria)
McAlister 2001b	DM is a combination of patient education, provider use of practice guidelines, appropriate consultation, and supplies of drugs and ancillary services.	Databases: Medline, EMBASE, CINAHL, SIGLE, Cochrane (1966~2000) Key words: case management, comprehensive health care, disease management, health service researches, home care services, clinical protocol, etc	12 studies included (RCTs) Inclusion criteria: randomized trial, more than 50 participants, impact of disease management on death, myocardial infarction, or admission rates.
McAlister 2001a	No explicit statement (only the words: comprehensive, multidisciplinary disease management)	Databases: Medline, EMBASE, CINAHL, SIGLE, Cochrane (1966~1999) Key words: case management, comprehensive health care, disease management, health service researches, home care services, clinical protocol, etc	11 studies included (RCTs) Inclusion criteria: randomized trial, effect of outpatient-based heart failure management programs on mortality or hospitalization rate, comprehensive DM system
***Qualitative review***			
Yu 2006	DM was operationally defined as a program that used multiple interventions in a systematic manner to manage HF across different health-care delivery system	Databases: Medline, EMBASE (1995~2005), Cochrane Controlled Trial Registry Key words: DMP, cardiac failure or heart failure, readmission and rehospitalization	21 studies included (21 RCTs) Inclusion criteria: patients with HF, hospital admission and mortality, mean age more than 60, detailed description of DM
Jerant 2005	DM is a systematic, population-based approach to identify persons at risk, intervene with specific programs of care, and measure clinical and other outcomes	Databases: Medline, PsychINFO, CINAHL, Cochrane (1966~2004) Key words: disease management, case management, telemedicine, home care services, home nursing, home care services, hospitalization, etc	33 studies included (RCTs) Inclusion criteria: RCT, DM program, results for patients with HF separately from those other diseases, telemedicine element
Ara 2004	DM is a systematic population-based approach to identify persons at risk, implement detailed programs of care, measure outcomes of interest, and achieve continuous quality improvement.	Databases: Medline, HealthSTAR, Cochrane, International Pharmaceutical abstracts (1966~2002) Key words: disease state management, disease management, intervention, quality improvement, managed care, health maintenance organization, etc	20 studies included (6 RCTs, 3 CTs, 11 before-after studies without control) Inclusion criteria: implementation of intervention, managed care settings, specific disease states (congestive heart failure, hypertension, hyperlipidemia-CAD)

**COPD & asthma**			
***Meta-analysis***			
Lemmens 2009	DM is a concept by which care delivery is coordinated through the integration of several components across the entire delivery system and the application of tools specifically designed for population in question	Databases: Medline and Cochrane Library (1995~2008) Key words: disease management, disease state management, delivery of integrated health care, comprehensive health care, patient care planning, primary health care, etc	36 studies included (28 RCTs, 8 before-after studies with control) Inclusion criteria: multiple interventions, patients aged over 16, control (usual care) or single intervention, objective measure of outcome, methodological quality
Peytremann-Bridevaux 2008	DM, a multidisciplinary approach proposed to enhance the quality and cost-effectiveness of health care for chronic conditions, has been defined as “an approach to patient care that emphasized coordinated, comprehensive care along the continuum of disease and across health care delivery system”	Databases: Medline, EMBASE, CINHAL, PsycINFO, Cochrane database (inception~2006) Key words: COPD, intervention relating to disease management, study design	13 studies included (9 RCTs, 1 before-after study with control, 3 before-after studies without control) Inclusion criteria: adult patients with COPD, fulfilling the operational definition of DM, not including inpatients only
Adams 2007	CCM identifies essential elements involving the community and health system and including self-management support, and clinical information systems.	Databases: Medline (1966~2005), CINAHL (1982~2005), Cochrane (2005) Key words: disease management, case management, chronic disease, self-care, self-management, patient education, lung diseases, obstructive, etc	32 studies included (26 RCTs, 5 CTs, 7 before-after studies with control) Inclusion criteria: interventions with at least 1 CCM components, with a control or comparison group (or outcome measured at two points), relevant outcomes
Talyor 2005	No explicit statement (only the words: nurse led chronic DM)	Databases: 16 English language databases (e.g., CINHAL, Cochrane, etc), 8 Dutch citation database (1980~2005) Key words: nursing care, community health nursing, patient discharge, outpatient clinic, home care service, community service, patient education, etc	9 studies included (RCTs) Inclusion criteria: clinical service intervention and package of care for managing COPD, nurse led, coordinated or delivered, randomized controlled trial
Sin 2003	DM is an approach to coordinate resources across the health care system with the aim of fostering community of care and increasing patients’ knowledge and control over their chronic disease.	Databases: Medline, Cochrane (1980~2002) Key words: no specific indications about DM	8 studies included (RCTs) Inclusion criteria: disease management programs (any combination of patient education, enhanced follow-up, self-management session)
***Qualitative review***			
Maciejewski 2009	DM programs are implemented to enable better disease control by supporting the practitioner/patient relationship and a plan of care to prevent exacerbations and complications.	Databases: Medline, EMBASE, CINAHL, PsychInfo, Cochrane database (1986~2008) Key words: asthma, managed care programs, disease management, case management, patient care team, comprehensive health care	27 studies included (5 RCTs, 7 before-after studies with control, 12 before-after studies without control, 3 only after studies with control) Exclusion criteria: children, patient education only, in-patient setting, opinion-based
Steuten 2009	The aim of DM programs is to improve processes and outcomes of care whilst making a more efficient use of scarce health care resources, or even generate cost savings (e.g., DMAA or CCM)	Databases: Medline, Cochrane database (1995~2007) Key words: disease management, disease state management, integrated delivery of health care, comprehensive health care, patient care planning, etc	17 studies included (14 RCTs, 2 CTs, 1 before-after study with control) Inclusion criteria: interventions more than two, studies with control or comparing group, relevant process or end outcomes evaluated
Niesink 2008	Chronic DM programs are interventions designed to manage or prevent a chronic condition using a systematic approach to care, with the potential use of multiple treatment modalities	Databases: Medline, EMBASE (1995~2006) Key words: COPD, pulmonary emphysema, chronic bronchitis, quality of life, health status, health status indicator, RCT, etc	10 studies included (10 RCTs) Inclusion criteria: RCT, clinical diagnosed COPD patients stable, outpatient integrated care, duration at least 8 weeks, measuring QOL
Steuten 2007	DM is a system of coordinated healthcare interventions and communications for people with conditions in which self-care efforts are significant.	Databases: Medline, and Cochrane database (2005~2006) Key words: six key components of DM in addition to disease management defined by DMMA, asthma, etc	8 studies included (3 RCTs, 4 before-after studies, one retrospective database study) Exclusion criteria: not compassing the whole continuum of care, single component of DM

**Diabetes**			
***Meta-analysis***			
Knight 2005	DM is programs that use a systematic approach of care and include more than 1 intervention component.	Databases: Medline, HealthSTAR, Cochrane, (1987~2001) Key words: disease state management, disease management, patient care team, patient care planning, primary nursing care, etc	24 studies included (19 RCTs, 5 CTs,) Inclusion criteria: adult patients, objective measurement of disease management, sufficient information to measure the effect of an intervention on at least 1 outcome, experimental or quasi-experimental study design
Noris 2001	DM is an organized, proactive, multicomponent approach to healthcare delivery that involves all members of a population with a specific diseases entity.	Databases: Medline, ERIC, CINAHL, HealthSTAR (1966~2000) Key words: case management, disease management, care model, shared care, primary health care, medical specialists, etc	27 studies included (5 RCTs, 1 CT, 5 cohort studies, 15 before-after studies with control, 3 other studies) Inclusion criteria: primary investigation, conducted in market economics, information on one or more outcomes of interest, all types of comparative studies

**Depression**			
***Meta-analysis***			
Neumeyer-Gromen 2004	DM is a multidisciplinary, dynamic care model that strives for continuous quality improvement. DM is a population-based care strategy and only assigned for highly prevalent chronic diseases.	Databases: Medline, PSYCLIT, PSYNDEX, EMBASE, Cochrane, BMJ database, etc (1966~2002) Key words: disease management program, depression management program, cost-effectiveness	10 studies included (RCTs) Inclusion criteria: complete DM programs with all components, randomized controlled study, adult patients above 18 years old
Badamgarav 2003b	DM is an intervention designed to manage or prevent a chronic condition by using a systematic approach to care and potentially employing multiple treatment modalities.	Databases: Medline, HealthSTAR, Cochrane (1987~2001) Key words: disease management, disease state management, patient care team, patient care planning, primary nursing care, case management, critical pathway, primary health care, etc	19 studies included (18 RCTs, 1 before-after study with control,) Inclusion criteria: complete DM programs with all components, randomized controlled study, adult patients above 18 years old

**Rheumatoid arthritis**			
***Meta-analysis***			
Badamgarav 2003a	DM is a multidisciplinary intervention to deliver by a team of health care professionals, providing a systematic approach to care, and including a patient education component.	Databases: Medline, HealthSTAR, EMBASE, Cochrane (1966~2001) Key words: disease management, patient care team, patient care planning, primary nursing care, case management, critical pathways, etc	11 studies included (8 RCTs, 3 CTs) Inclusion criteria: target population, evaluation of a DM intervention, patients’ functional status, RCT or quasi-experimental design

**Multiple diseases**			
***Meta-analysis***			
Tsai 2005	The CCM is a primary care-based framework aimed at improving the care of patients with chronic illness. The model integrates a number of elements into a plausible package designed to foster more productive interactions between prepared, proactive teams and well-informed motivated patients.	Databases: Medline, Cochrane for systematic reviews, and Medline for more recent individual studies (1993~2003) Key words: 4 diseases (asthma, congestive heart failure, depression, diabetes), 6 elements of CCM (23 items)	112 randomized or non-randomized trial studies included (27 asthma, 21 congestive heart failure, 33 depression, 31 diabetes) Inclusion criteria: randomized or nonrandomized controlled studies, interventions tested (6 elements), outcomes of interest (clinical outcome, QOL, process of care)
Krause 2005	DM is defined as a system of coordinated healthcare interventions and communications for populations with conditions in which patient self-care efforts are significant.	Databases: Medline, DMMA’s LitFinder (1995~2003) Key words: disease management, economic, outcomes	67 studies included (34 experimental, 10 quasi-experimental, 23 before-after studies), (28 heart, 28 asthma, 11 diabetes) Inclusion criteria: chronic disease types (asthma, diabetes, heart disease), economic outcome measures (medial cost, hospital admission, clinic visit, emergency visit), DM interventions (self-management, nurse-management, team-management), study method
Weingarten 2002	DM is an intervention designed to manage or prevent a chronic condition using a systematic approach to care and potentially employing multiple treatment modalities.	Databases: Medline, HealthSTAR, Cochrane (1987~2001) Key words: disease state management, disease management, patient care team, patient care planning, primary nursing care, case management, critical pathway, primary health care, continuity of patient care, guidelines, etc	102 experimental or quasi-experimental studies included (26 diabetes, 25 depression, 10 asthma, 9 congestive heart failure, 9 rheumatoid arthritis, 7 hypertension, 7 COPD, etc ) Inclusion criteria: guideline or systematic approach, experimental or quasi-experimental study, estimation of at least one relevant measure of program effects
***Qualitative review***			
Ofman 2004	DM is an intervention designed to manage or prevent a chronic condition using a systematic approach to care and potentially employing multiple treatment modalities. The same as Weingarten	Databases: Medline, HealthSTAR, Cochrane (1987~2001) Key words: disease state management, disease management, patient care team, patient care planning, primary nursing care, case management, critical pathway, primary health care, continuity of patient care, guidelines, etc	102 experimental or quasi-experimental studies included (22 diabetes, 25 depression, 9 heart failure, 9 rheumatoid arthritis, 8 asthma, 7 hypertension, 6 COPD, etc ) Inclusion criteria: pertain to chronic diseases, objective measurement of processes or outcomes, a systematic approach to care, experimental or quasi-experimental study, estimation of at least one relevant measure of program effects

DM: disease management, DMAA: Disease Management Association of America, CCM: chronic care model, RCT: randomized controlled trial, CT: controlled trial, HF: heart failure, COPD: chronic obstructive lung disease

Despite the diversity of the definition of DM, several key components were mentioned in the definitions of DM as follows: systematic, comprehensive, population-based, multi-components, coordinated healthcare, specific disease entity, continuous quality improvement, multidisciplinary, practice guidelines, patient and/or provider education, and so on (Table 1). As is shown in Table 2, in each article, several key words were used for searching relevant articles for meta-analyses or systematic reviews on DM as follows: disease management, case management, comprehensive health care, health service research, multidisciplinary care, guidelines, home care services, patient care planning, primary care nursing, and so on.

**Table 2:** Characteristics of analysis and main results

Article	Diseases, interventions and outcome measures	Main results	Conclusion
**CHD**			
***Meta-analysis***			
Göhler 2006	Diseases: congestive heart failure Interventions: multidisciplinary approach, interventions centered on specific health professionals Outcomes: all-cause mortality, all-cause hospitalization rate	Mortality: Difference=3% (CI 1~6%) Rehospitalization: Difference=8% (5~11%) Factors for heterogeneity: severity of disease, proportion of beta-blocker as baseline, country, duration of follow-up, mode of post discharge contact	DM programs have the potential to reduce morbidity and mortality for patients with congestive HF. The benefit of intervention depends on age, severity of disease, guideline-based treatment, and DM program modalities.
Roccaforte 2005	Diseases: heart failure (HF) Interventions: multidisciplinary approach, interventions centered on specific health professionals Outcomes: mortality, hospitalization rate	Mortality reduction: OR=0.80 (CI 0.69~0.93) Hospitalization rates for all-cause: OR=0.76 (0.69~0.94) for HF 0.58 (0.50~0.67) ACE-1 therapy rate: OR=1.48 (1.20~1.83) Different DM approaches were equally effective.	DM program reduce mortality and hospitalizations in HF patients. The choice of a specific program depends on local health services characteristics, patient population, and resource available.
Whellan 2005	Diseases: HF Interventions: clinic follow up by a physician extender with cardiology or primary care physician supervision, home nursing follow up, telephone follow up by a physician extender Outcomes: all-cause hospitalization, QOL, mortality, cost	All-cause hospitalization: significant reduction with heterogeneity QOL: more consistent improvement Mortality: no difference in mortality with some exceptions Interventions using clinic follow-up by a cardiologist, home visit, or telephone follow-up significantly reduced all-cause hospitalization.	DM is an intervention that could significantly decrease hospitalization for patients with HF. Due to differences in the types of strategies and the variety of health care settings, further studies of DM programs with multiple participating centers are required.
Phillips 2005	Diseases: HF Interventions: complex components of DM, hospital discharge planning Outcomes: readmission, mortality, and the combined endpoint of mortality and hospitalization	Readmission: OR=0.91 (CI 0.72~1.16) Mortality rate: OR=0.80 (0.57~1.06) Combined endpoint: 0.88 (0.74~1.04) Suspected better outcomes for programs with hospital discharge planning and post-discharge follow-up (without statistical comparison).	DM with specialist nurse-led HF clinics would be a promising strategy or effective alternative whose benefit may be optimized by programs with a homogeneous structure and components that are delivered with consistency.
Gonseth 2004	Diseases: HF Interventions: types of DM programs with home visits, out-patient visits to a clinic, patient longer follow-up Outcomes: hospital readmission for HF or other cardiovascular causes, all-cause readmission, readmission and mortality	Readmission in RCTs for HF or CVD : RR=0.70 (CI 0.62~0.79), for all-cause: RR=0.88 (0.79~0.97) Combined event of readmission or death: RR=0.82 (0.72~0.94) The magnitude of DM program benefits reported by non-randomized studies was more than double that reported by randomized studies.	DM programs are effective at reducing re-admissions among elderly patients with HF. Their effectiveness is close to that observed in clinical trials evaluating drugs for HF. However, the relative effectiveness of types of healthcare delivery within the DM program is not known.
McAlister 2001b	Diseases: coronary heart disease Interventions: multidisciplinary DM program Outcomes: reinfarction, all causes mortality, admission to hospital, process of care, QOL, costs	Prescription of efficacious drugs: RR=2.14 (CI 1.92~2.38) Improvement of risk factor profiles: significant improvement (moderate rage) All cause mortality: RR=0.91 (0.79~1.04) Recurrent myocardial infarction: RR=0.94 (0.80~1.10) Admission: 0.84 (0.76~0.94) QOL or functional status: better outcomes in 3 studies Costs: savings in 2 studies	DM programs improve process of care, reduce admissions to hospitals and enhance quality of life or functional status in patients. The programs’ impact on survival and recurrent infarctions, their cost-effectiveness, and mix of components remain uncertain.
McAlister 2001a	Diseases: HF Interventions: comprehensive, multidisciplinary DM program Outcomes: hospitalization rate, all cause mortality, medication, QOL, costs	Cost: saving in 7 of 8 trials reporting costs reported Prescribing: beneficial effects Hospitalization rate: RR=0.87 (CI 0.79~0.96) All cause mortality: RR=0.94 (0.75~1.19) Specialized follow-up by a multidisciplinary team led a significant reduction in hospitalization, while telephone contact failed to find any benefits.	DM programs for the care of HF involving specialized follow up by a multidisciplinary team reduce hospitalization and appear to be cost saving. Data on mortality are inconclusive. Further studies are needed to establish the incremental benefits of the different elements of DM programs.
***Qualitative review***			
Yu 2006	Diseases: HF Interventions: Education, counseling, self-care support, optimized medication, early attention to clinical deterioration, vigilant follow-up Outcomes: hospital readmission, mortality, combined events, QOL, costs	Significant effects (follow-up more than 3 months): hospital readmission 53% (10/19), mortality 3% (3/13), combined event 62% (8/13), QOL 50% (4/8), cost reduction 88% (7/8) Suggested factors for effective DM program (not statistically significant): case management, multi-disciplinary team, counseling by allied health, optimized medical therapy, exercise counseling, home visit	This study defines precisely the characteristics of the care team and the organization content and delivery method of the DM program which are crucial to enhance the discharge outcomes of older people with HF..
Jerant 2005	Diseases: HF Interventions: HFDM incorporating telemedicine, 4 types of interventions Outcomes: hospitalization, emergency visit, mortality, QOL, costs	Hospitalizations and emergency visits: significant reduction Mortality, costs and QOL: varied among fewer studies which examined them There was no significant improvement in any outcomes among less severe disease and /or in health systems with preexisting proactive approach.	HFDM programs incorporating telemedicine can reduce acute care utilization by severely affected patients, but their impact on other outcomes is unproven. Less symptomatic patients and those cared for in well-organized health systems do not appear to benefit from HFDM.
Ara 2004	Diseases: cardiovascular disease (congestive heart failure, hypertension, hyperlipidemia and/or coronary artery disease) Interventions: multiple health care professionals, patient and physician education, intensive drug therapy, lifestyle modification, close patient monitoring Outcomes: not specified	A variety of interventions demonstrate some effectiveness in improving to the 3 disease states. While all 5 studies for CHF appeared to be successful, 3 studies among 9 studies of hypertension and 6 studies of heperlipidemia-CAD were unsuccessful. A few studies employed a fully experimental design and posed significant limitations.	A number of cardiovascular DM strategies reported promising results. Many of the multidisciplinary CH DM programs were more complex than were those for hypertension and hyperlipidemia-CAD, due to the nature and severity of the disease.

**COPD and asthma**			
*** Meta-analysis***			
Lemmens 2009	Diseases: asthma, COPD Interventions: patient education, professional education, expansion or revision of professional roles, and/or case management Outcomes: QOL, hospital admission, healthcare utilization, satisfaction, emergency visit	QOL score: difference =-4.59 (CI -8.34~-0.83) Hospital admission: OR=0.58 (0.40~0.83) Emergency department visit: difference=-4.59 (CI -8.34~-0.83) Process and knowledge: improvement in most studies Lung function: no improvement Healthcare utilization and satisfaction: ambiguous	DM programs in asthma and COPD shows improvement in QOL and reduction in hospitalization in multiple interventions. No effects on emergency department visits were found. Improvement in process was found in most studies. There was not consistent improvement in outcome indicators.
Peytremann-Bridevaux 2008	Diseases: COPD Interventions: 2 or more components (e.g., physical exercise, self-management, structured follow-up), 2 or more health care professionals involved in patient care and patient education Outcomes: all-cause mortality, lung function, exercise capacity, QOL, acute exacerbations, health care use, etc	Exercise capacity: Difference=32.2 (CI 4.1~60.3) Mortality: OR=0.85 (CI 0.54~1.36) Significant effects: Lung function 14% (1/7), QOL 73% (8/11), symptoms 43% (3/7), health care use 70% (7/10)	COPD DM programs modestly improved exercise capacity, health-related QOL, and hospital admission, but not mortality.
Adams 2007	Diseases: COPD Interventions: self-management, delivery system design, decision support, clinical information system	Hospitalization: RR=0.79 (0.66~0.94) Emergency department visit: RR=0.58 (CI 0.42~0.79) Mortality: RR=0.58 (CI 0.26~1.29) Significant improvement: knowledge 56% (5/9), QOL 20% (2/10), length of hospital stay in 7 studies Cost reduction: 11% to 70% in 10 studies Dyspnea, lung function,: no clinically significant improvement	Pooled data, evaluating the efficacy of CCM components in COPD, demonstrated that patients with COPD who received interventions with 2 or more CCM components had lower rates of hospitalizations and emergency/unscheduled visits and a shorter length of stay compared with control groups.
Talyor 2005	Diseases: COPD Interventions: nurse led chronic DM, brief interventions, long term or intensive interventions Outcomes: survival, healthcare utilization, ADL, QOL of patients and carers	Mortality: OR=0.85 (CI 0.58~1.26) Emergency attendance, knowledge: possible improvement Patients’ QOL, psychological wellbeing, disability, pulmonary function, symptoms: no difference detected	There is little evidence to date to support the widespread implementation of nurse led management interventions for COPD, but the data are too spare to exclude any clinical relevant benefit or harm arising from such interventions.
Sin 2003	Diseases: COPD Interventions: DM program, patient education, enhanced follow-up Outcomes: mortality, hospitalization rate, QOL	Mortality: RR=0.63 (CI 0.38~1.04) Hospitalization: RR=0.86 (0.68~1.08) SGRQ score: -2.5 (14.8~ -0.1)	DM programs appear to improve health status of patients, but may not meaningfully impact on hospitalization and mortality. However, this finding may reflect differences in the core of DM strategies across various studies.
***Qualitative review***			
Maciejewski 2009	Diseases: asthma Interventions: patients and/or providers education, assessment and monitoring of patients, non-physician providers involvement Outcomes: health outcome, process, hospitalization, emergency–department visits, etc	Statistically significant effects: clinical outcomes (e.g., symptoms) 55% (16/29), medications 57% (16/28), process (e.g., inhaler technique) 55% (16/30), economic outcomes (e.g., hospitalization) 58% (45/77), patient-reported outcomes (e.g., quality of life) 47% (22/47)	Few well-designed studies with rigorous statistical evaluation have been conducted to evaluate DM interventions for adults with asthma. Current evidence is insufficient to recommend any particular DM model or interventions.
Steuten 2009	Diseases: COPD Interventions: self-management, delivery system design, decision support, clinical information systems	Statistically significant effects: disease-specific knowledge 80% (4/5), QOL 53% (8/15), decrease in health care utilization 50% (7/15) Symptoms and function: equivalent Coordination of care: mixed Total costs:: no significant change	Identifying cost effective multi-components COPD programs remains a challenges due to scarce methodologically sound studies that demonstrate significant improvements on process, intermediate and end results of care.
Niesink 2008	Diseases: COPD Interventions: multidisciplinary care team, clinical pathway, case management, self-management or patient education	Statistically significant improvement: QOL 50% (5/10) Clinically relevant improvement: QOL 70% (7/10) in intervention groups, 40% (4/10) in control groups	All chronic DM projects for people with COPD involving primary care improve quality of life. In most of the studies, aspects of chronic DM were applied to a limited extent. Quality of RCTs was not optimal.
Steuten 2007	Diseases: asthma Interventions: educational (e.g. self-management, or disease-specific knowledge), professional (e.g., changing performance or adherence to guidelines), organizational (e.g., improving the continuity of care) interventions	Statistically significant effect: hospitalization or exacerbations 71% (5/7), total costs (1/1), patient satisfaction (1/1) Educational related process: mixed Symptoms or lung function: no significant change QOL: no significant change (0/3) Organizational effect: mixed	There is accumulating circumstantial evidence that DM programs reduce resource utilization. But, the generalizability of results remains uncertain.

**Diabetes**			
*** Meta-analysis***			
Knight 2005	Diseases: diabetes mellitus Interventions: interventions of a systematic approach Outcomes: glycated hemoglobin, serum lipids, systolic blood pressure, hospital admission, screening for retinopathy, etc	Glycated hemoglobin control: -0.49 (CI -0.56~ -0.41) Monitoring glycemic level: significant increase Screening for retinopathy: significant increase Foot screening and referral: improvement Foot care: significant decrease Screening for nephropathy: inconclusive	Diabetes DM programs can improve glycemic control to a model extent and can increase screening for retinopathy and foot complications.
Noris 2001	Diseases: diabetes mellitus Interventions: DM and case management Outcomes: glycemic level, lipid level, BMI, QOL, knowledge, satisfaction, utilization, monitoring and screening	Glycemic level: -0.5% (CI -1.35~ -0.1) Monitoring, screening: significant improvement Annual examination 7.7% (2.7~45.0) Self-monitoring, QOL: significant improvement Lipid level, blood pressure, cost: inconclusive	This evidence for DM is applicable to adults with diabetes in managed care organization and community clinics in the US and Europe. Case management is effective both when delivered in conjunction with DM and when delivered with one or more additional educational, reminder or support intervention

**Depression**			
***Meta-analysis***			
Neumeyer-Gromen 2004	Diseases: depression Interventions: complete DM program (evidence-based practice guidelines, self-management education, etc) Outcomes: depression severity, QOL, employ status, satisfaction, adherence to treatment regimen, cost-effectiveness ratio	Depression severity: RR=0.75 (CI 0.70~0.81) Adherence to medication: RR=0.59 (0.46~0.75) Overall appropriate care: RR=0.77 (0.70~0.85) CU ratio: $9,051 - $49,500 per QALY Patient satisfaction: RR=0.57 (0.37~0.87) QOL: insufficient data available Employment: significant holding in 1 study	DM program significantly enhance the quality of care for depression. Costs are within the range of other widely accepted public health improvement.
Badamgarav 2003b	Diseases: depression Interventions: multimodal DM program Outcomes: depression symptom, physical functioning, social and health status, satisfaction, healthcare utilization, hospitalization, cost, etc	Depression symptom: ES=0.33 (CI 0.16~0.49) Patient satisfaction: ES=0.51 (0.33~0.68) Detection of derpession : ES=0.66 (0.22~1.1) Adequate prescription : ES=0.44 (0.30~0.59) Patients’ adherence : ES=0.36 (0.17~0.54) Other outcomes : inconclusive (not significant)	DM appears to improve the detection and care of patients with depression.

**Rheumatoid arthritis**			
*** Meta-analysis***			
Badamgarav 2003 a	Diseases: rheumatoid arthritis Interventions: DM program, duration, number of units of interventions Outcomes: functional status	Functional status: ES=0.27 (CI -0.01~0.54) Functional status (HAQ): ES=0.16 (-0.13~0.44) Functional status in long intervention duration: ES=0.49 (0.12~0.86)	There were limited data to support or refute the effectiveness of DM programs in improving functional status in patients with RA.

**Multiple diseases**			
*** Meta-analysis***			
Tsai 2005	Diseases: asthma, congestive heart failure, depression, diabetes Interventions: CCM, delivery system design, self-management support, decision support, clinical information system, community resources, healthcare organization Outcomes: clinical outcome, process of care	Over all Clinical outcome: ES=-0.23 (CI -0.31~ -0.15) QOL: ES=0.11 (0.02~0.21) Process of care: RR=1.19 (1.10~1.28) Delivery system design Clinical outcome: ES=-0.21 (CI -0.40~ -0.02) Process of care: RR=1.16 (1.01~1.34) Self-management Clinical outcome: ES=-0.22 (CI -0.38~ -0.05) Decision support Process of care: RR=1.29 (1.08~1.54)	Interventions that contain at least 1 CCM element improve clinical outcomes and process of care (and to a lesser extent, QOL) for patients with chronic illness.
Krause 2005	Diseases: heart disease, asthma, diabetes Interventions: DM interventions, self-management, nurse-management, team-management Outcomes: medial cost, hospital admission, clinic visit, emergency visit	Effect size (unbiased): 0.311 (CI 0.272~0.350) The general linear model analysis indicated a statistically significant difference in disease severity (p<0.05), but not in the types of diseases and interventions.	DM programs are more economically effective with severely ill enrollees, and DM interventions are most effective when coordinated with the overall level of severity. The findings can be generalized.
Weingarten 2002	Diseases: 118 diseases (diabetes, depression, asthma, congestive heart failure, rheumatoid arthritis, hypertension, COPD, etc) Interventions: DM program, education, feedback, reminders, financial incentives). Outcomes: disease control, provider adherence to guidelines, patient disease control.	Interventions directed at providers on disease control Provider education: ES=0.35 (CI 0.19~0.51) Provider feedback: ES=0.17 (0.1~0.25) Provider reminder: ES=0.22 (0.1~0.37) adherence to guidelines Provider education: ES=0.44 (0.19~0.68) Provider feedback: ES=0.61 (0.28~0.93) Provider reminder: ES=0.52 (0.35~0.89) Interventions directed at patients on disease control Provider education: ES=0.24 (0.07~0.40) Provider feedback: ES=0.27 (0.17~0.36) Provider reminder: ES=0.40 (0.26~0.54)	All studied interventions were associated with improvements in provider’s adherence to practice guidelines and disease controls. The type and number of interventions varied greatly, and future studies should directly compare different type of intervention to find the most effective.
*** Qualitative review***			
Ofman 2004	Diseases: the same as Weingarten Interventions: patient education, provider education, multidisciplinary team/shared care, provider feedback, provider reminder, patient financial, organizational financial, provider financial Outcomes:	Substantial improvement (percentage of comparison): depression 48% (41/86), hyperlipidemia 45% (5/11), CAD 36% (24/69), asthma 25% (9/36), rheumatoid arthritis 24% (7/29), back pain 16% (3/19), COPD 9% (2/22), chronic pain 8% (1/12). Statistically significant outcomes: patient satisfaction 71% (12/17), patient adherence 47% (17/36), disease control 45% (33/74), provider adherence 40% (14/35), patient knowledge 31% (4/13), morbidity 29% (7/24), mortality 24% (4/17), QOL 16% (5/31), other utilization 16% (4/25), costs: 14% (1/7), emergency visit 11% (1/9), hospitalization 11% (3/28)	DM programs were associated with marked improvements in many different processes and outcomes of care. Few studies demonstrated a notable reduction in costs.

### 3.2 Effectiveness and Efficiency of DM Programs

a. Overall Studies

Table 2 shows the characteristics of DM programs, main results and conclusions among articles (i.e., 20 meta-analyses and 8 qualitative systematic reviews). Table 3 shows the summary of important outcomes in DM programs. Details of improvement in each article and in each item are shown in Table 2.

**Table 3:** Summary of important outcomes in disease management programs

Article	Mortality	Health outcome	Process	QOL	Satis-faction	Knowledge or life-style	Health services	Costs
**CHD**								
*** Meta-analysis***								
Göhler 2006	○	–	–	–	–	–	○	–
Roccaforte 2005	○	–	○	–	–	–	○	–
Whellan 2005	?	–	?	Δ?	–	–	○	?
Phillips 2005	×	–	–	Δ	–	–	×	?
Gonseth 2004	○*	–	–	–	–	–	○	Δ
McAlister 2001b	×	○	○	Δ	–	–	○	Δ?
McAlister 2001a	×	–	Δ	Δ?	–	–	○	?
***Qualitative review***								
Yu 2006	?	–	–	Δ	–	–	Δ	Δ
Jerant 2005	?	–	–	?	–	–	Δ	?
Ara 2004								
Congestive heart failure	–	Δ?	Δ?	–	–	–	Δ?	–
Hypertension	–	Δ	Δ?	Δ?	–	Δ?	Δ?	Δ
Hyperlipidemia	–	Δ?	Δ?	–	–	Δ?	–	–

**COPD and asthma**								
*** Meta-analysis***								
Lemmens 2009	–	×	Δ?	○	Δ?	Δ?	○	–
Peytremann-Bridevaux 2008	×	○	–	Δ	–	?	Δ	–
Adams 2007	×	×	–	Δ	–	Δ	○	Δ
Talyor 2005	×	×	–	×	–	Δ?	Δ?	–
Sin 2003	×	–	–	○	–	–	×	–
*** Qualitative review***								
Maciejewski 2009	–	Δ	Δ	Δ	–	–	Δ	–
Steuten 2009	×	×	?	?	–	×	Δ	?
Niesink 2008	–	–	–	Δ	–	–	–	–
Steuten 2007	–	×	?	Δ	Δ?	–	Δ	Δ?

**Diabetes**								
*** Meta-analysis***								
Knight 2005	–	○	Δ	Δ?	–	Δ?	Δ?	–
Noris 2002	–	○	○	Δ?	Δ?	?	Δ?	?

**Depression**								
*** Meta-analysis***								
Neumeyer-Gromen 2004	–	○	○	Δ	○	–	–	○
Badamgarav 2003b	–	○	○	×	○	–	×	×

**Rheumatoid arthritis**								
*** Meta-analysis***								
Badamgarav 2003a	–	○	–	–	–	–	–	–

**Multiple diseases**								
*** Meta-analysis***								
Tsai 2005	–	○	○	○	–	–	–	–
Krause 2005	–	–	–	–	–	–	–	○^#^
Weingarten 2002								
Overall	–	–	○	–	–	○	–	–
CHD	–	–	○	–	–	○	–	–
Diabetes	–	–	○	–	–	○	–	–
Depression	–	–	○	–	–	○	–	–
COPD	–	–	–	–	–	×	–	–
Rheumatoid arthritis	–	–	○	–	–	?	–	–
*** Qualitative review***								
Ofman 2004	?	?	Δ	–	Δ	Δ	?	?

Health outcomes (e.g., morbidity, disability, function), process (e.g., compliance or adherence to guidelines), health services (e.g., hospitalization or admission); ○ significant improvement by meta-analysis, Δ improvement by qualitative review × insignificant, Δ? suggestive but limited, ? unclear or ambiguous, * re-admission or death, # economic measure integrating costs and health services, ^–^ not available

The item most frequently evaluated was health services (e.g., hospitalization, admission, etc) (79%, 22/28), followed by QOL (75%, 21/28), health outcomes (e.g., physiological and functional status, disability, etc) (57%, 16/28), healthcare process (e.g., adherence to guidelines, screening frequency, etc) (57%, 16/28), costs (57%, 16/28), mortality (54%, 15/28). The proportion of articles evaluating knowledge or life-style change, and satisfaction was 36% (10/28) and 21% (6/28), respectively.

The proportion of articles which observed improvement with considerable evidence (the symbol ○ means meta-analysis and △ means qualitative review) was the highest for process (69%, 11/16), followed by health services (63%, 14/22), QOL (57%, 12/21), satisfaction (50%, 3/6), knowledge or life-style (30%, 5/16), health outcomes (51%, 9/16), costs (38%, 6/16) and mortality (20%, 3/15). As to mortality, statistically significant results were observed only in the area of coronary heart disease (CHD).

If improvement with limited evidence (indicated by △? for qualitative review) was included, this proportion increased, and ranged from 100% (satisfaction) to 20% (mortality). In particular, the proportion in satisfaction, QOL, health services, and knowledge or life-style increased respectively to 100%, 81%, 81% and 70%. On the other hand, if improvement was limited only to results with statistically significance by meta-analysis (○symbol), the proportion decreased and ranged from 50% (health outcome) to 10% (knowledge or life-style).

b. Coronary Heart Diseases

Seven meta-analyses (Gohler et al., 2006; Roccaforte et al., 2005; Whellan et al., 2005; Phillips et al., 2005; Gonseth et al., 2004; McAlister et al., 2001a, 2001b) and 3 qualitative reviews (Yu et al., 2006; Jerant et al., 2005; Ara, 2004) evaluated DM programs for CHD, including hypertension and hyperlipidemia (Tables 2 and 3). In addition, 1 meta-analysis 49) evaluated DM programs for CHD among multiple diseases.

The proportion of articles which observed improvement with considerable evidence was the highest for health services (80%, 8/10), followed by process (67%, 4/6), satisfaction (50%, 3/6), health outcomes (50%, 1/2), knowledge or life-style (50%, 1/2), QOL (43%, 3/7), costs (38%, 3/8) and mortality (33%, 3/9). As to mortality, statistically significant results by meta-analyses were observed only recently (Gohler et al., 2006; Roccaforte et al., 2005). The item of satisfaction was not evaluated.

If improvement with limited evidence was included, the proportion in health outcome, knowledge or life-style, health services, QOL and process increased to 100% (2/2), 100% (2/2), 90% (9/10), 86% (6/7) and 83% (5/6), respectively.

c. COPD

Five meta-analyses (Lemmens et al., 2009; Peytremann-Bridevaux et al., 2008; Adams et al., 2007; Taylor et al., 2005; Sin et al., 2003) and four qualitative reviews (Maciejewski et al., 2009; Steuten et al., 2009; Niesink, 2007; Steuten et al., 2007) evaluated DM programs for COPD (Tables 2 and 3). In addition, 1 meta-analysis49 evaluated DM programs for COPD among multiple diseases.

The proportion of articles which observed improvement with considerable evidence was the highest for QOL (78%, 7/9), followed by health services (75%, 6/8), process (40%, 2/5), knowledge or life-style (33%, 2/6), costs (33%, 1/3) and health outcomes (29%, 2/7). Those for mortality and satisfaction were 0% (0/5) and 0% (0/2), respectively. If improvement with limited evidence was included, the proportion in satisfaction, health services, knowledge or life-style, costs increased to 100% (2/2), 88% (7/8), 67% (4/6), and 67% (2/3), respectively.

d. Diabetes

Two meta-analyses (Knight et al., 2005; Noris et al., 2002) evaluated DM programs for diabetes mellitus (Tables 2 and 3). In addition, 1 meta-analysis (Weingarten et al., 2002) evaluated DM programs for diabetes mellitus among multiple diseases.

The proportion of articles which observed improvement with considerable evidence was the highest for process (100%, 3/3) and health outcomes (100%, 2/2), followed by knowledge or life-style (67%, 2/3). Those in QOL, satisfaction, health services and costs were 0%. Mortality was not evaluated. If improvement with limited evidence was included, the proportion in knowledge or life-style, QOL, satisfaction and health services increased to 100%.

e. Depression

Two meta-analyses (Neumeyer-Gromen et al., 2004; Badamgarav et al., 2003b) evaluated DM programs for depression (Tables 2 and 3). In addition, 1 meta-analysis (Weingarten et al., 2002) evaluated DM programs for depression among multiple diseases. The proportion of articles which observed improvement with considerable evidence was 100% in health outcomes (2/2), process (3/3), satisfaction (2/2) and knowledge or life-style (1/1), followed by QOL (50%, 1/2) and costs (50%, 1/2). That in health services was 0% (0/1). Mortality was not evaluated.

f. Rheumatoid Arthritis

One meta-analysis (Badamgarav et al., 2003a) evaluated DM programs for rheumatoid arthritis (Tables 2 and 3). In addition, 1 meta-analysis (Weingarten et al., 2002) evaluated DM programs for rheumatoid arthritis among multiple diseases. Improvement was observed for health outcomes (1/1), process (1/1), and knowledge or life-style (1/1). Five items (i.e., mortality, QOL, satisfaction, health services and costs) were not evaluated.

g. Multiple Diseases

Three meta-analyses (Tsai et al., 2005; Krause, 2005; Weingarten et al., 2002) and 1 qualitative review (Ofman et al., 2004) evaluated DM programs on multiple diseases (Tables 2 and 3).

The proportion of articles which observed improvement with considerable evidence was 100% in process (3/3), QOL (1/1), satisfaction (1/1) and knowledge or life-style (2/2), followed by health services (50%, 1/2) and costs (50%, 1/2). That in mortality and health services was 0% (0/1).

### 3.3 Features of Interventions in DM Programs

While the types of interventions in DM programs were summarized in Tables 2, the results related to specific interventions by meta-analyses and qualitative reviews are shown in Table 4. The proportion of articles which examined effect or impact of specific interventions in DM programs was 43% (12/28).

**Table 4:** Evaluation of intervention features, quality of studies, and economics for disease management

Article	Intervention features	Quality of studies	Economics
**CHD**			
***Meta-analysis***			
Göhler 2006	Multidisciplinary team and personal post-discharge contact were more effective and suggested as factors explaining heterogeneity in re-hospitalization between studies.	–	–
Roccaforte 2005	High quality studies and multidisciplinary programs appeared to be more consistently associated with a beneficial effect on mortality and health failure related re-hospitalization rates.	The quality of each study was evaluated according to component approach, examining randomization, blinding and so on. Thirty percent of studies were decided to be of high quality.	–
Whellan 2005	Interventions using clinic follow up by specialist, home visit, or telephone follow up significantly decreased all-cause hospitalization.	–	Although most of studies reported a cost for providing the intervention, it only reflected estimates of direct personal expenses. Indirect expenses were not included.
Phillips 2005	Interventions with hospital discharge planning were more effective in readmission rate.	In assessing methodological quality, the Jadad score for each study was calculated. The median Jadad score was 3.5. Sixty-seven percent of studies were of high quality.	Only three programs reported complete data for the cost of care (initial hospital care, intervention costs, out patient care, and charges for readmissions). The potential savings was observed, but not significant.
Gonseth 2004	–	The study quality was assessed by the Jadad scale for randomized controlled trial. Only 11 of 27 trials attained a core of 3 on the scale. Among 27 non-randomized trials, no study adjusted for confounding factors.	Thirteen studies assessed the cost of DM programs. Only several studies considered intervention costs besides healthcare costs.
McAlister 2001b	–	–	Only three trials described the costs of interventions. Two reported cost savings, but none performed formal cost-effectiveness analysis.
McAlister 2001a	Multidisciplinary team providing specialized follow-up reduced the risk of hospitalization.	–	Only one trial reported cost saving. There is no detailed cost description.
***Qualitative review***			
Yu 2006	Characteristics of effective DM programs were analyzed by case-control like analysis. The difference was observed in several items including counseling in hospital by allied health and exercise counseling, but none of them were statistically significant.	–	Seven of eight effective programs were indicated to be cost saving. However, there is no information on costs, except cost per case.
Jerant 2005	–	The quality of studies was assessed according to the User’s Guide to Medical Literature. Only eight of 33 trials were judged to be of acceptable.	Reduction of acute care costs and medical care charges were mentioned based on systematic reviews. There is no detailed information on costs.
Ara 2004	–	–	One study indicated cost-effectiveness based on blood pressure reduction. The other three studies mentioned expenditure per capita, medical care costs and cost of anti-hypertensive therapy, but there was no detailed information on costs.

**COPD & asthma**			
*** Meta-analysis***			
Lemmens 2009	Triple interventions (patient-related, professional-directed and organizational interventions) including case management showed significant difference in quality of life, although double interventions did not. Double interventions including a pharmacist showed significant difference in quality of life, although triple interventions did not. However, a qualitative comparison suggested more significant effects of triple rather double interventions.	Study quality was assessed with the Health Technology Assessment, Disease Management instrument (0 to 100 points). Studies of inferior quality (below 50 points) were excluded. Forty-two percent of studies were evaluated as good quality.	–
Peytremann-Bridevaux 2008	–	The quality of trials was assessed using 3 different instruments (Jadad score, qualitative categories by Cochrane Collaboration. and Health Technology Assessment, Disease Management instrument). The mean Jadad score was 2.4. Studies with high quality in other scales were less than half.	–
Adams 2007	The relative risk of emergency visits and hospitalizations were significantly low for multi-component studies.	The US Preventive Task Force criteria were used. Only one study was evaluated as good and four studies were as fair among 20 RCTs.	Among 4 trials reporting costs, three demonstrated a range of 34% to 70% reduction in health care costs in the intervention groups, predominantly because of reduced hospitalizations.
Talyor 2005	–	The Delphi list and the Jadad criteria were used. Most of 9 trials had potential methodological limitations.	–
Sin 2003	–	The scoring system was not used to evaluate the quality of the trials. The authors restricted the analysis to trials with randomization, placebo-control, blind ascertainment of end point, and so on.	–
***Qualitative review***			
Maciejewski 2009	–	The quality of study was examined in terms of study design, intervention description, and statistical adjustment. The studies’ quality was poor in these respects.	–
Steuten 2009	–	The methodological quality of the articles was evaluated with the Health Technology Assessment, Disease Management instrument (0 to 100 points). The overall mean score was 67.6. Forty-seven percent of studies were of good quality.	Three studies, presenting cost data, showed difference observed (e.g., prescription costs, hospitalization related costs). However, none of them reported significant changes in total costs.
Niesink 2008	–	The 11 criteria were used to assess methodological quality. The average score was 5.8. The proportion of studies with high score (i.e., more than six) was 60%.	–
Steuten 2007	–	The methodological quality of the articles was evaluated with the Health Technology Assessment, Disease Management instrument (0 to 100 points). The overall mean score was 60.0. Only three studies showed good quality.	In one study, significant decrease in annual total costs was reported. However, using total costs as a single primary outcome measure poses a threat to the validity of outcome.

**Diabetes**			
***Meta-analysis***			
Knight 2005	–	–	–
Noris 2001	–	Studies met the minimum quality standard of the evidence-based Guide to Community Preventive Services, method.	One study showed no difference in average cost between intervention and control groups after 2 years. The other cost-benefit study showed incremental benefit cost ratio of 1.86. Both studies were classified as good.

**Depression**			
***Meta-analysis***			
Neumeyer- Gromen 2004	–	The validity assessment of each study was conducted on the basis of the Cochrane Collaboration Handbook. Except for three studies with quality of A/B, B and B/C, all other studies were those of best quality (A).	Based on 6 cost-effectiveness/cost-utility analysis, overall cost-utility ratios ranged between $9,051 and $49, 500 per quality adjusted life year. The studies were evaluated by NHS EED economists as mostly valid and reliable.
Badamgarav 2003b	–	–	All three programs measured total health services cost associated with treatment and indicated that program participants incurred higher costs. But the effect was not statistically significant.

**Rheumatoid arthritis**			
***Meta-analysis***			
Badamgarav 2003a	Based on the number of units of interventions, the studies offering equal or less than 6 units of interventions were associated with higher effect, although estimates did not reach statistical significance.	–	–

**Multiple diseases**			
*** Meta-analysis***			
Tsai 2005	Four elements of the CCM (delivery system design, self-management support, decision support, and clinical information system) were associated with better outcomes and processes.	In assessing methodological quality, the Jadad score for each study was calculated. Among 93 RCTs, only 32% scored 3, and none scored higher than 3. However, double blinding is rarely possible in studies of organizational interventions.	–
Krause 2005	Statistically significant difference of effect size was observed by DM interventions (e.g., team-managed) and disease severity, but the former was not found after the latter was taken into consideration.	–	As direct economic measures, 4 items (i.e., medical cost, hospital admissions or readmissions, physician office or clinic visit, and emergency department visits) were used. The individual effect size values were averaged and included as one construct.
Weingarten 2002	Education, feedback and reminder for provider, as well as education, reminder and financial incentives, were all associated with improvement in provider adherence to guidelines and/or patient disease control.	–	–
***Qualitative review***			
Ofman 2004	–	The quality of clinical trials was assessed using criteria described by Jadad.	Utilization and cost-related outcomes showed benefit in relatively few studies.

DM: disease management, DMAA: Disease Management Association of America, CCM: chronic care model, RCT: randomized controlled trial, CT: controlled trial, HF: heart failure, COPD: chronic obstructive pulmonary disease

Multidisciplinary team approaches (Gohler et al., 2006; Roccaforte et al., 2005; McAlister et al., 2001a) were the most frequently indicated intervention which were related to health outcomes (e.g., mortality or morbidity) and health services utilization (e.g., hospitalization). Also, multi-component programs (Lemmens et al., 2009; Adams et al., 2007; Tsai et al., 2005) were mostly shown or suggested to be effective in QOL, health services, and other items, in both usual DM and CCM programs.

Besides these interventions, other interventions were as follows: clinical follow-up by specialists (Whellan et al., 2005), home visit (Whellan et al., 2005), hospital discharge planning or post-discharge follow-up (Gohler et al., 2006; Phillips et al., 2005), counseling in hospital by allied health professionals (Steuten et al., 2009), and education or reminder for providers and patients (Weingarten et al., 2002).

On the other hand, while disease severity as a target of DM programs played an important role (Krause, 2005), high quality of studies (Roccaforte et al., 2005) and long term interventions (Roccaforte et al., 2005; Taylor et al., 2005; Badamgarav et al., 2003b) were important factors for evaluating effectiveness.

### 3.4 Economics

The economic evaluation of DM programs was reported in 16 articles (Tables 3 and 4). The proportion of articles with considerable evidence, indicating some form of favorable effects, was 38% (6/16). If articles with limited evidence were included, the proportion increased to 50% (8/16).

However, as is shown in Table 4, there were few comprehensive economic revaluations and their systematic reviews. While there was no, or very limited, information on costs in articles (McAlister et al., 2001a; Yu et al., 2006; Jerant et al., 2005; Ara, 2004; Steuten et al., 2007; Ofman et al., 2004). costs for interventions, rather than healthcare costs, were rarely or insufficiently described, even if costs were reported (Whellan et al., 2005; Adams et al., 2007; Steuten et al., 2009; Badamgarav et al., 2003b; Krause, 2005). Only one article (Neumeyer-Gromen et al., 2004) showed the cost-effectiveness/cost-utility ratios, as an economic summary measure, which varied from $9,051 to $49,500 per QALY.

### 3.5 Study Design and Quality of Studies Reviewed

The main types of study design of studies reviewed among the articles were randomized controlled trials (RCTs), controlled trials (CTs), cohort studies and before-after studies (Table 1). As is shown in Table 1, the proportion of articles, which included only RCTs (or experimental studies) and RCTs or CTs (or quasi-experimental studies) were 43% and 64%, respectively. The former proportion varied from 100% to 19%.

Table 4 shows information on the results of reviewing the quality of studies included in each article. The proportion of articles evaluating quality of studies was 61% (17/28). While the Jadad score (1996) was used in about one third of quality evaluations (Phillips et al., 2005; Gonseth et al., 2004; Peytremann-Bridevaux et al., 2008; Taylor et al., 2005; Taais et al., 2005; Ofman et al., 2004), other criteria such as the HTA Disease Management (Steuten et al., 2004), the Cochrane Collaboration Handbook (2006) and the evidence-based Guide to Community Preventive Services-method (Briss et al., 2000) were also used. The proportion of studies with high or good quality, according to the criteria used, varied widely form 20% to 80%.

## 4. Discussion

The synthesis of meta-analyses and systematic reviews on DM programs showed considerable evidence of their effectiveness on outcomes from healthcare outcomes, health process and health services, to knowledge, satisfaction, QOL and costs (Tables 2-4). Even if only meta-analyses were examined, similar results were obtained. However, as to mortality, evidence is very limited and inconclusive. Two recent meta-analyses (Gohler et al., 2006; Roccaforte et al., 2005) showed statistically significant mortality reduction. These results were mostly shared among subgroups of target diseases, such as CHD, COPD, diabetes and depression. The evidence of DM programs for rheumatoid arthritis is very limited.

An earlier synthesis report about DM programs (Velasoc-Garrido et al., 2003), based on only four systematic reviews available at that time, was unable to draw definitive conclusions about diverse outcomes of DM programs. Incorporating meta-analyses and systematic reviews over the past seven years, this synthesis could analyze the evidence on effectiveness and efficiency more deeply and comprehensively. However, the results of this study should be carefully examined for application to healthcare decision making.

First, DM programs vary widely in their structure, and are hard to describe with a single definition, since they usually contain many components. In this analysis, there existed no common definition of DM, and a considerable variation was observed (Tables 1 and 2). Also, diverse key words for searching literature on DM were differently mixed and used (Table 2). These different definitions and search strategies may lead to the inclusion of different studies and yield different results. While these results depend on the specific operational definition of DM, the effects of DM programs among different articles seem to be relatively similar (Table 3). However, there is an urgent need to develop a consensus on the more systematic or common concept and classification of DM programs, to allow for more reliable and valid analyses and comparisons.

Second, it is difficult to confirm the relative effectiveness and efficiency of the types, components or interventions of DM programs. In this synthesis, each article adopted its own components or interventions of DM programs (Tables 2 and 3). Most of these components and interventions were closely related to the components mentioned in the definitions of the Disease Management Association America (DMAA) (Disease Management Association America, 2003) and the Chronic Care Model (Bodenheimer et al., 2002).

These articles compared different components or their combinations, arbitrarily rather than systematically or comprehensively. The analysis of components indicated the statistically significant impact of the following components or interventions: multidisciplinary team interventions, clinical follow-up by a specialist, home visit or telephone follow-up, discharge planning and post-discharge follow-up, delivery system design, self-management, decision support, and so on (Tables 2-4).

This evidence obtained is closely related to the six components that appear to influence of effectiveness of DM after detailed analyses of Medicare demonstration projects after their failure (Brown, 2009). Also, new developments for chronic disease care, such as medical home (Rosenthal, 2008), care coordination (Boult et al., 2010) and transitional care (Naylor et al., 2004) shared a lot of components with DM programs in these articles reviewed, although the emphasis on components and their structure are very different among these approaches and DM programs. On the other hand, as to each component adopted by existing DM programs, there is evidence based on systematic reviews in self-management (Chodosh et al., 2005; Warsi et al., 2004; Blaiss, 2004, Monninkhof et al., 2003), care management (Windham et al., 2003), case management (Norris et al., 2002; Ferguson et al., 1998), multidisciplinary care (Philbin, 1999; McAlister et al., 2004) and integrated care (Owens et al., 2005).

Although these findings give an insight into the effective components of DM programs, the evaluation of components or interventions, as well as their combinations, is still in its infancy. It is not possible to draw conclusions about essential components or their ideal combinations for DM programs. A future challenge is the need to develop and implement more systematic and comprehensive analyses on components of DM programs.

Third, careful examination about the types of study design and their quality is the basis of evaluation of DM programs. In evaluating effectiveness of healthcare, RCT is recognized as a gold standard of a hierarchy of strength of evidence (Guyatt et al., 2002). The main types of study design for evaluating DM programs in this synthesis were randomized controlled trials (RCTs) and controlled trials (CTs), which comprised 43% to 64% of studies evaluating DM programs among the articles reviewed. The proportion of studies with high and good quality varied from 20% to 80%.

These figures are not satisfactory to assure the validity of studies evaluated by the articles. However, they should be cautiously interpreted. The assessment of complex multi-component interventions and diverse measures of outcomes are methodological challenges unique to organizational or community interventions like DM programs. Although well-conducted RCTs provide the most reliable evidence, these are not always feasible for interventions in this situation. Also, RCTs may increase the internal validity, while decreasing the external validity. Corresponding to this challenge, the guide for allowing non-randomized controlled trials or observational studies is proposed by the Cochrane Effective Practice and Organization of Care Review Group (EPOC) (Cochrane Effective Practice and Organization of Care Review Group) and the Task Force on Community Preventive Services (Briss et al., 2000). Moreover, from the perspective of recent argument on comparative effectiveness study, practical trials or advanced observational studies would be more suitable for evaluation of DM programs (Dreyer et al., 2010).

The additional important issue related to study design is the unclear status of comparators. The studies in the reviews included in the articles used mostly compare DM programs with usual care. However, the organization and provision of usual care differs across and within healthcare systems. Therefore, precise components and interventions of usual care should be described and evaluated simultaneously. In evaluating DM programs, application of these criteria and consideration of comparators would increase feasibility and flexibility of evaluation and enable the evaluation to reflect the comprehensive view of effectiveness and efficiency of DM programs.

Forth, the most controversial outcomes in DM programs are mortality and cost-effectiveness. In this analysis, although the effectiveness of DM programs has been shown on health outcomes, process and services, only two meta-analyses demonstrated statistically significant mortality reduction in coronary heart diseases (Gohler et al., 2006; Roccaforte et al., 2005). In COPD, four meta-analyses failed to demonstrate significant decrease in mortality, although relative risks of mortality were less than 1 (Peytremann-Bridevaux et al., 2008; Adams et al., 2007; Taylor et al., 2005; Sin et al., 2003). There may be several explanations. Most analyses did not have a large enough sample size to detect a mortality reduction. Also, the follow-up period of the studies was relatively short. The average follow-up of most studies is less than 12 months. A large study population with long-term follow-up would be needed to evaluate actual impact of DM programs on mortality.

The number of meta-analyses and systematic reviews of economic evaluation of DM programs are relatively limited (Tables 3-4). The proportion of articles with favorable effects in costs or cost-effectiveness was 38% in this analysis. However, there were few articles which evaluated formally and comprehensively economic results. Economic evaluation is defined as the comparative analysis of alternative courses of action in terms of both their costs and consequences (Drummond et al., 2005). Since most studies of economic evaluation on DM programs focused only on costs, they are classified as partial evaluation (e.g., cost analysis), rather than full economic evaluation (e.g., cost-effective and cost-benefit analysis) (Drummond et al., 2005).

Furthermore, where costs were examined, undefined or very limited cost items, such as medical care costs, were evaluated in the articles. The implementation of DM programs requires substantial investments in development of a program, and additional human or organizational costs for implementing it (Kesteloot, 1999). A recent review (Goetzel et al., 2005) on return on investment (ROI) of DM programs reported a positive ROI in congestive heart failure and multiple diseases, but the range and detail of costs and benefits was unclear and limited under a financial point of view. Therefore, the cost savings or cost-effectiveness of DM programs still remained undetermined, and formal full economic evaluations are needed.

Finally, the generalizability and transferability of the findings in this synthesis has not been examined for applying them within and across countries. While DM programs have continued to evolve and mature over time and places, with failure and success, they have diffused from the US to other countries such as the UK (Departement of Health, 2002; Mason et al., 1999), Germany (Busse, 2004; Boseken, 2003), the Netherlands (Vrijhoef et al., 2001), Canada (Tsasis et al., 2008) and developing countries (WHO, 2002). In fact, many studies evaluating DM programs have been already carried out in other countries. For example, in a comprehensive systematic review (Roccaforte et al., 2005; Gonseth et al., 2004; Weingarten et al., 2002), while about a half of the studies came from the US, the other half came from the UK, the Netherlands, Sweden, Australia, Canada, Finland, Argentina, New Zealand, Italy, Israel, and so on. Therefore, much effort should be made towards examining generalizability or transferability of evidence about DM programs, according to the characteristics and context of the healthcare system in each country.

In summary, there is considerable evidence on the effectiveness and efficiency of DM programs, and several components or interventions in DM programs were suggested to be effective. However, further research is needed to examine which type or component of DM programs works best and efficiently. Also, new emerging approaches for care coordination of chronic diseases should be integrated or added to the DM approach.
